# Ultrahypofractionated linac-based radiotherapy of primary prostate cancer - early results of an Austrian nationwide registry study

**DOI:** 10.1016/j.ctro.2026.101242

**Published:** 2026-07-21

**Authors:** Natalee Annon-Eberharter, Johannes Berchtold, Angela Ginestet, Georg Gruber, Lukas Kocik, Oxana Komina, Tanja Langsenlehner, Elisabeth Nechvile, Christoph Resl, Falk Röder, Samuel M. Vorbach, Ludmila Zahlbruckner, Frank Wolf

**Affiliations:** a Department of Radiation Oncology, Paracelsus Medical Private University (PMU), Salzburg, Müllner Hauptstraße 48, Salzburg 5020, Austria; b Institute of Research and Development of Advanced Radiation Technologies (radART), Paracelsus Medical Private University (PMU), Salzburg, Austria; c Department of Radiation Oncology, Medical University of Innsbruck, Comprehensive Cancer Center Innsbruck (CCCI), Innsbruck, Austria; d Department of Radiation Oncology, Ordensklinikum Linz Barmherzige Schwestern, Linz, Austria; Medical Faculty, Johannes Kepler University Linz, Linz, Austria; e Department of Radiation Oncology, Wiener Gesundheitsverbund Klinik Hietzing, Wien, Austria; f Medical University of Graz, Neue Stiftingtalstraße 6, 8010 Graz, Austria; g Division of Radiotherapy-Radiation Oncology, Karl Landsteiner University of Health Sciences, Krems an der Donau, Austria; h Institute for Radiooncology, Wiener Gesundheitsverbund, Klinik Ottakring, Vienna, Austria

**Keywords:** Prostate SBRT, SABR, UHFX, Hypofractionation, Registry, Austria

## Abstract

**Background and purpose:**

Ultrahypofractionated radiotherapy (UHFX) has emerged as an attractive alternative to normo- and moderately hypofractionated regimens for localized prostate cancer. However, its broader adoption remains limited by technical challenges and concerns regarding genitourinary toxicity. We established a nationwide Austrian registry of low and intermediate risk localized prostate cancer patients treated with linac-based stereotactic body radiation therapy (SBRT) to provide real-world data about UHFX treatment conventions, toxicity, oncologic outcomes, and patient-reported quality of life (QoL).

**Methods:**

This is a multicenter, registry-based prospective study. Eligibility was restricted to patients with low- and intermediate-risk prostate cancer. Patient characteristics, toxicity (CTCAE v5.1), urinary function (IPSS), and QoL (EPIC-26) were prospectively reported in a web-based database.

**Results:**

Between September 2022 and April 2026, 191 patients were enrolled, of whom 169 were included in the final analysis. Median follow-up was 14 months (IQR 7–24). NCCN risk distribution consisted of 23.7% low-risk, 48.5% favorable intermediate-risk, and 27.8% unfavorable intermediate-risk disease. All patients received 40 Gy in five fractions. Treatment was delivered on alternating weekdays in 61.5% and on consecutive weekdays in 38.5% of patients. Median PSA declined from 7.74 ng/mL at baseline to 0.63 ng/mL at 12 months. No biochemical recurrence was observed during the first year of follow-up. Grade 1–2 genitourinary toxicity peaked at treatment completion and declined thereafter, while grade ≥ 3 toxicity remained uncommon (<1%). Gastrointestinal toxicity remained low throughout follow-up, with no grade ≥ 3 gastrointestinal toxicity observed.

**Conclusion:**

In this nationwide real-world cohort, UHFX delivered in five fractions was well tolerated and showed favorable early oncological outcomes with low rates of severe toxicity across academic and non-academic institutions, consistent with results from randomized phase III trials.

## Highlights

• An Austrian nationwide registry provides real world data on UHFX usage, oncological outcome, toxicity and quality of life.
• 169 low and intermediate risk patients received linac-based radiotherapy delivering 40Gy in 5 Fractions
• Severe genitourinary and gastrointestinal toxicity was uncommon. One year biochemical recurrence free survival was 100%.
• Nationwide implementation of prostate SBRT was feasible and reproduced phase III trial outcomes.

## 1 Introduction

Ultrahypofractionated radiation therapy (UHFX), also known as stereotactic body radiation therapy (SBRT), usually refers to fractionation regimens with fraction doses exceeding 5Gy [1]. This approach aims to exploit the intrinsic property of prostate cancer cells to exhibit higher sensitivity to large doses per fraction. [2]. This biological property reflected in their low α/β ratio remains integral regardless of the technical advances in radiation delivery. However, the boundaries within which the linear quadratic model remains reliable, are not well defined and its robustness when very few fractions are applied has recently been challenged [3].

While moderately hypofractionated radiation therapy (mHFX) has been widely adopted in recent years, UHFX adoption seems to progress at a slower pace, even though it is endorsed by international guidelines [4], [5] and provides substantial advantages in terms of patient comfort and cost [6], [7].

Randomized phase III data have shown that UHFX is non-inferior to mHFX in terms of biochemical disease-free survival [8], [9]. However, the body of evidence lags behind the considerable amount of data supporting both mHFX and normofractionated radiotherapy. Slight increases in genitourinary (GU) and gastrointestinal toxicity (GI) [10] as well as discussions about the technical requirements in terms of intrafraction motion control, margins, rectal spacers and urethral sparing [11] have raised concerns about UHFX implementation, particularly in smaller, non-academic institutions.

At the time of registry initiation, UHFX was sparingly used in comparison to mHFX. This nationwide registry was established to document treatment patterns, image-guidance strategies, androgen deprivation therapy (ADT) use, toxicity, prostate-specific antigen (PSA) response, and patient-reported quality of life (QoL) in patients treated with linac-based UHFX. In addition, outcomes were evaluated in the context of previously published randomized UHFX trials. The registry provides prospective multicenter data on the early implementation of prostate SBRT in routine clinical practice across Austria.

## 2 Methods

### 2.1 Study design and patient eligibility

This prospective multicenter registry study was conducted across seven Austrian radiation oncology centers. Consecutive patients treated with linac-based ultrahypofractionated radiotherapy (UHFX) for localized prostate cancer were eligible for enrollment following written informed consent. The study was approved by the local ethics committee.

Eligible patients had histologically confirmed adenocarcinoma of the prostate with NCCN very low-, low-, favorable intermediate- or unfavorable intermediaterisk disease and a WHO performance status of 0–2. Patients with very low-risk disease were eligible if active surveillance was considered unsuitable.

Exclusion criteria included prior pelvic radiotherapy, inflammatory bowel disease, or active secondary malignancy within the preceding 10 years unless treated with curative intent and in sustained remission.

### 2.2 Treatment planning and delivery

Simulation CT was performed with full bladder and empty rectum preparation, including enema when clinically indicated. Multiparametric MRI was fused with the planning CT for target delineation. The use of gold fiducials and rectal spacers was permitted according to institutional practice.

All patients received 40 Gy in five fractions delivered using IMRT- or VMAT-based linac techniques. Fractionation schedules consisted of either consecutive daily treatment or alternating weekday delivery (2–3 fractions per week). Posterior planning target volume (PTV) margins ranged from 3 to 6 mm according to institutional motion-management protocols.

Short-term androgen deprivation therapy (ADT) was recommended for patients with unfavorable intermediate-risk disease in accordance with current guideline recommendations. ADT use in favorable intermediate- and low-risk disease was left to physician discretion.

### 2.3 Image guidance and motion management

Daily image guidance was mandatory for all treatments. Cone-beam CT (CBCT) was used in all participating centers, while selected centers additionally employed ExacTrac-based intrafraction monitoring or RayPilot HypoCath™ real-time electromagnetic tracking. The choice of image-guidance modality and motion-management strategy was left to institutional preference.

### 2.4 Clinical outcomes and follow-up assessments

Clinical data were prospectively entered into a web-based registry platform and stored in an SQL database hosted by Salzburger Landeskliniken (SALK). Physician-reported toxicities were graded according to Common Terminology Criteria for Adverse Events (CTCAE) version 5.1. Lower urinary tract symptoms were assessed using the International Prostate Symptom Score (IPSS). Patient-reported quality-of-life outcomes were evaluated using the Expanded Prostate Cancer Index Composite short form (EPIC-26).

Baseline assessments were performed before treatment initiation. Follow-up assessments were scheduled at treatment completion, 3 months, 6 months, 12 months, and annually thereafter. Assessments obtained within ±14 days of the predefined follow-up interval were accepted for analysis. The first follow-up assessment was restricted to within 7 days from last the radiotherapy fraction.

### 2.5 Statistical analysis

Statistical analysis was primarily descriptive. Continuous variables are reported as median and interquartile range (IQR), while categorical variables are presented as counts and percentages. Longitudinal toxicity and patient-reported outcome data were analyzed according to available-case methodology without inclusion of missing values. PSA kinetics, toxicity, IPSS, and EPIC-26 outcomes were evaluated descriptively across predefined follow-up intervals.

Statistical analyses were performed using Excel LTSC (Microsoft Corporation, Redmond, WA, USA) and Python (Python Software Foundation, Wilmington, DE, USA).

## 3 Results

Between September 2022 and April 2026, 191 patients were prospectively enrolled across seven Austrian radiation oncology centers. Median center accrual was 29 patients (range 11–49). Twenty-two patients were excluded from the final analysis because of treatment modification, inability to tolerate fiducial or catheter placement, leaving 169 patients for analysis.

Median follow-up was 14 months (IQR 7–24). Median patient age was 74 years (IQR 69–79). 23.7% of patients had NCCN low-risk disease, while 48.5% and 27.8% had favorable and unfavorable intermediate-risk disease, respectively. MRI fusion biopsy was performed in 83.4% of patients, and 21.3% underwent PSMA PET/CT staging. Baseline patient and disease characteristics are summarized in Table 1.

**Table 1:** Patient characteristics.

Variable	
Participating centers	7
Total patients recruited	191
Analyzed cohort	169
Median center accrual (range)	29 (11–49)
Median age (IQR), years	74 (69–79)
**Median baseline PSA, ng/mL (IQR)**	7.74 (5.06–10.66)
**Medan positive biopsy cores (IQR)**	35% (23–47%)
**NCCN risk group**	
Low risk	40 (23.7%)
fIR	82 (48.5%)
uIR	47 (27.8%)
**Gleason grade group (ISUP)**	
Grade Group 1 (3 + 3)	41 (24.3%)
Grade Group 2 (3 + 4)	107 (63.3%)
Grade Group 3 (4 + 3)	21 (12.4%)
Grade Group 4 (4 + 4)	0 (0%)
**Clinical T stage**	
T1a-c	113 (66.9%)
T2a-c	56 (33.1%)
**MRI fusion biopsy**	141 (83.4%)
**PSMA PET/CT staging**	36 (21.3%)
**Median follow-up (IQR), months**	14 (7–24)

Values are presented as n (%) unless otherwise indicated. IQR, interquartile range; PSA, prostate-specific antigen; NCCN, National Comprehensive Cancer Network; ISUP, International Society of Urological Pathology; fIR, favorable intermediate-risk; uIR, unfavorable intermediate risk.

ADT was administered in 39.1% of the overall cohort, including 17.5% of low-risk patients, 35.4% of patients with favorable intermediate-risk disease, and 63.8% of patients with unfavorable intermediate-risk disease, reflecting substantial variation in ADT utilization across risk groups.

All patients received a five-fraction regimen delivering 40 Gy to the clinical target volume without reported treatment interruptions. Treatment was delivered on alternating weekdays in 61.5% of patients and on consecutive days in 38.5%. All patients underwent image-guided radiotherapy. CBCT-based guidance was used in 73.4% of patients, while 30.8% additionally underwent ExacTrac-based intrafraction motion monitoring. Gold fiducials were implanted in 89.9% of patients, and two centers utilized RayPilot HypoCath™ real-time tracking in 16.6% of the cohort [12]. Rectal spacers were used in 14.2% of patients (13.6% gel spacer, 0.6% other spacer). Posterior PTV margins ranged from 3 to 6 mm, with 3 mm margins used in 62.1% of patients. Treatment-related characteristics are summarized in Table 2.

**Table 2:** Treatment and image-guidance characteristics.

Variable	Analyzed cohort (*n* = 169)
**Treatment delivery**	
Total dose	40 Gy in 5 fractions
Consecutive daily fractions	65 (38.5%)
Alternating weekdays (2–3 fractions/week)	104 (61.5%)
**ADT use**	
Any ADT use	66 (39.1%)
Low risk	7/40 (17.5%)
fIR	29/82 (35.4%)
uIR	30/47 (63.8%)
**Planning and target margins (Posterior PTV)**	
3 mm	105 (62.1%)
4 mm	38 (22.5%)
5 mm	25 (14.8%)
6 mm	1 (0.6%)
**Image guidance and motion management**	
CBCT-based IGRT	124 (73.4%)
ExacTrac intrafraction monitoring	52 (30.8%)
Gold fiducials	152 (89.9%)
RayPilot HypoCath™	28 (16.6%)
**Rectal spacer use**	
Gel spacer	23 (13.6%)
Other spacer	1 (0.6%)

Values are presented as n (%) unless otherwise indicated. Percentages may not total 100% because image-guidance modalities overlapped. ADT, androgen deprivation therapy; PTV, planning target volume; CBCT, cone-beam computed tomography; IGRT, image-guided radiotherapy; fIR, favorable intermediate-risk; uIR, unfavorable intermediate risk.

**Fig. 1:**
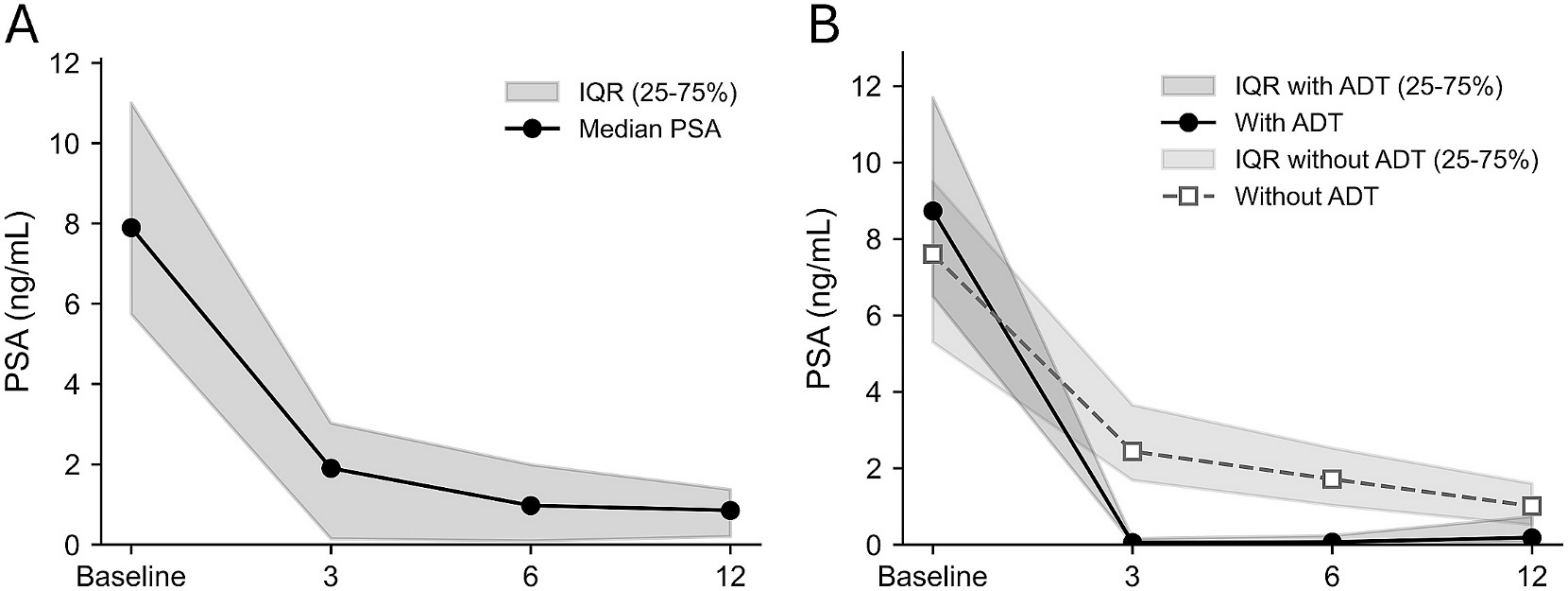
Median prostate-specific antigen (PSA) levels over time following ultrahypofractionated radiotherapy. PSA kinetics for (A) the overall cohort and (B) stratified by androgen deprivation therapy (ADT) use. Baseline PSA corresponds to the highest value recorded prior to treatment. Shaded areas indicate interquartile range (IQR).

Median baseline prostate-specific antigen (PSA) was 7.74 ng/mL (IQR 5.06–10.66). PSA levels declined rapidly following UHFX, with the greatest reduction observed within the first 3 months after treatment. At 12-month follow-up, median PSA was 0.63 ng/mL, corresponding to a 91.9% reduction from baseline. No biochemical recurrence, defined according to the Phoenix definition (nadir +2 ng/mL), was observed during the first year of follow-up (see Fig. 1A). Patients treated without androgen deprivation therapy (ADT) demonstrated a slower early PSA decline compared with patients receiving ADT, although PSA levels converged during later follow-up intervals (Fig. 1B).

**Fig. 2:**
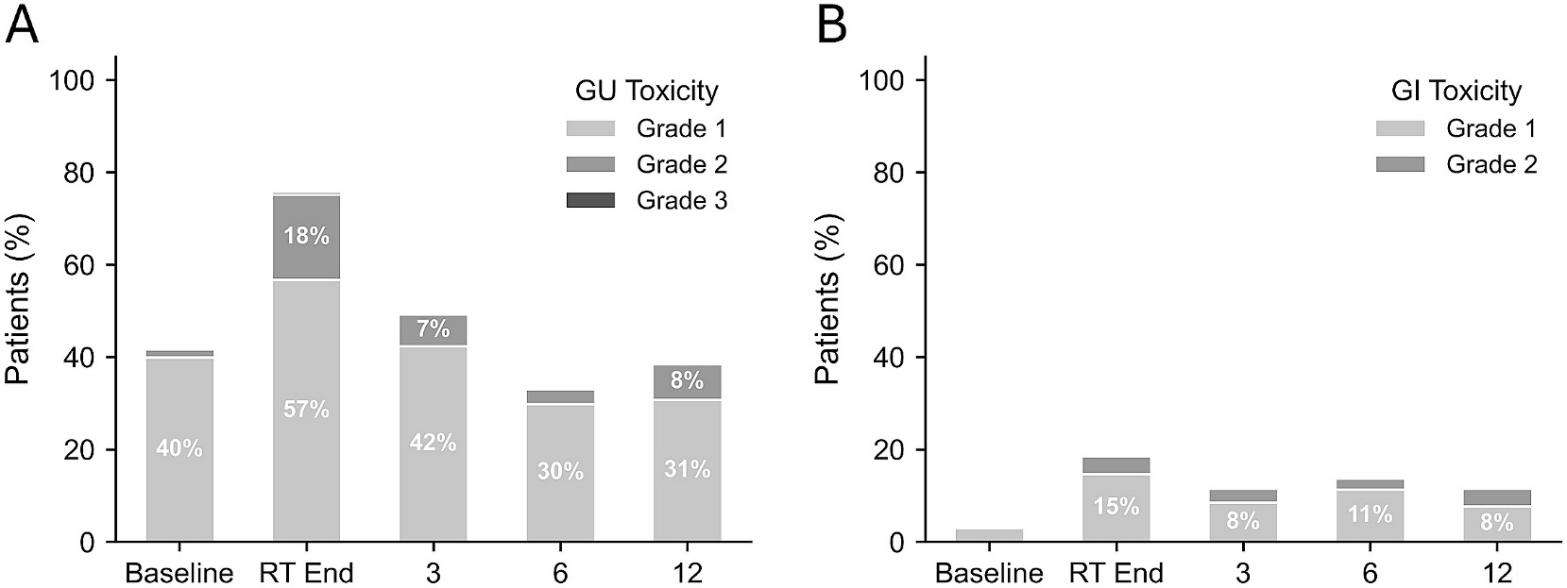
Physician-reported toxicity following ultrahypofractionated radiotherapy according to Common Terminology Criteria for Adverse Events (CTCAE) version 5.1. (A) Genitourinary (GU) toxicity. (B) Gastrointestinal (GI) toxicity.

Compliance with longitudinal patient-reported outcome assessment was high, with EPIC-26 and IPSS completion rates ranging from 89% to 100% across available follow-up intervals (Supplementary Table S1). UHFX was generally well tolerated. Grade 1–2 genitourinary (GU) toxicity peaked at treatment completion and was observed in 75.2% of patients. GU toxicity declined thereafter, with grade 1–2 events observed in 49.2% of patients at 3 months, 33% at 6 months, and 38.5% at 12 months. Grade 3 GU toxicity occurred in one patient (0.6%) at treatment completion, with no persistent grade ≥ 3 GU events during later follow-up (Fig. 2A).

Gastrointestinal (GI) toxicity remained low throughout follow-up. Grade 1–2 GI toxicity was observed in 18.4% of patients at treatment completion, 11.6% at 3 months, 13.7% at 6 months, and 11.5% at 12 months. No grade ≥ 3 GI toxicities were observed (see Fig. 2B).

**Supplementary Fig.1:**
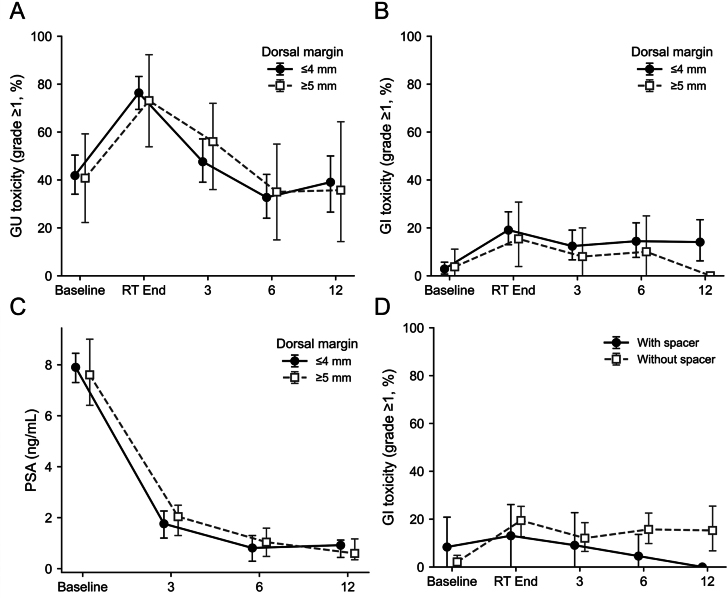
Exploratory subgroup analysis of planning target volume (PTV) dorsal margins and spacer use. (A) Genitouniary toxicity (GU), (B) gastrointestinal toxicity (GI) and (C) PSA kinetics in patients with ≤4 mm and ≥ 5 mm PTV margins. (D) gastrointestinal toxicity ≥1 of patients with and without spacer.

Given the variability in treatment approaches across participating centers, exploratory subgroup analyses were performed according to posterior PTV margin size and rectal spacer use. No significant differences in grade ≥ 1 GU or GI toxicity were observed between patients treated with posterior PTV margins of ≤4 mm and ≥ 5 mm at any assessed timepoint. PSA kinetics were also comparable between these groups, with no significant differences observed at baseline or during follow-up (Supplementary Fig. 1A-B)). Similarly, rectal spacer use (*n* = 24) was not associated with a significant reduction in grade ≥ 1 GI toxicity during the available follow-up period (Supplementary Fig. 1D).

**Fig. 3:**
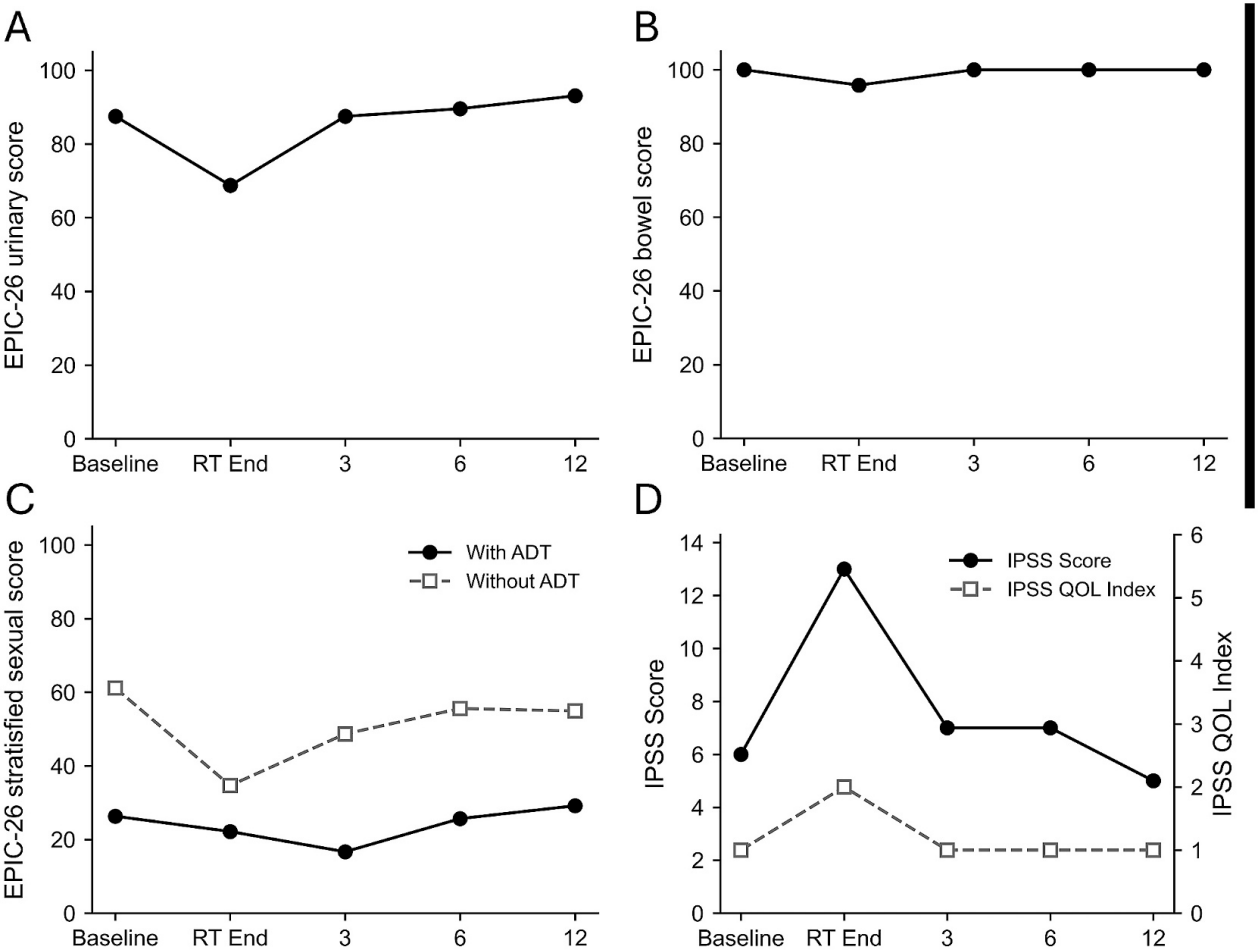
Patient-reported quality-of-life outcomes following ultrahypofractionated radiotherapy. Data are shown for (A) EPIC-26 urinary domain score, (B) EPIC-26 bowel domain score, (C) EPIC-26 sexual domain score stratified by androgen deprivation therapy (ADT) use, and (D) International Prostate Symptom Score (IPSS) and IPSS-related quality-of-life score over time.

EPIC-26 urinary domain scores declined at completion of radiotherapy and recovered rapidly to baseline levels from 3 months onward (Fig. 3A). EPIC-26 bowel domain scores remained largely stable throughout follow-up with only minor fluctuations over time (Fig. 3B).

Sexual domain scores declined at completion of radiotherapy with partial recovery during subsequent follow-up (Fig. 3C). When stratified by androgen deprivation therapy (ADT), patients receiving ADT reported substantially lower sexual function scores throughout follow-up compared with patients treated without ADT (Fig. 3C). In both groups, the greatest decline was observed at completion of radiotherapy, followed by recovery during subsequent follow-up.

Median International Prostate Symptom Score (IPSS) increased transiently at completion of radiotherapy and gradually returned toward baseline values during later follow-ups,. Similarly, the reported voiding-related quality of life (IPSS-related quality-of-life score) recovered to near-baseline levels by 12 months (Fig. 3D).

## 4 Discussion

This nationwide registry demonstrates that five-fraction UHFX can be implemented across a heterogeneous network of Austrian radiation oncology centers with low rates of severe toxicity and favorable early oncologic outcomes. The observed toxicity profile and PSA kinetics were consistent with previously published randomized phase III trials evaluating prostate SBRT [8], [9], [10], [13].

The registry also provides insight into how linac-based UHFX has been adopted across academic and non-academic institutions during the early implementation phase in Austria. In contrast to several previously published SBRT datasets frequently including multiple treatment platforms, including CyberKnife-based approaches [14], [15], the present registry exclusively evaluated linac-based treatment delivery. Given the broad availability of contemporary linac systems and their increasing integration into routine practice, these findings may provide a more representative overview of prostate UHFX implementation. The registry demonstrated some variability in technical treatment approaches, reflecting the real-world implementation of prostate UHFX across participating centers. Exploratory subgroup analyses did not identify significant differences in GU or GI toxicity or PSA kinetics between patients treated with posterior PTV margins of ≤4 mm and ≥ 5 mm.

Rectal spacer use was also not associated with a significant reduction in GI toxicity during the available follow-up period. This finding should be interpreted with caution given the low overall incidence of GI toxicity and the relatively small proportion of patients treated with a spacer.

The role of UHFX in unfavorable intermediate-risk and high-risk prostate cancer remains less well established than in low- and favorable intermediate-risk disease. Patients with unfavorable intermediate-risk (uIR) disease were included in the present registry because they constituted a substantial proportion of patients enrolled in both HYPO-RT-PC and PACE-B [8], [9]. Although patients with Gleason score 4 + 3 disease were excluded from PACE-B, many trial participants would currently be classified as uIR based on the presence of multiple intermediate-risk factors. In contrast, high- and very high-risk patients were underrepresented in these randomized trials and were therefore not included in the present registry.

Median age in our registry was 74 years (IQR 69–79), slightly higher than the PACE-B cohort (median approximately 70 years), reflecting the real-world nature of this multicenter cohort. In contrast to randomized trials in which ADT use was not permitted, our protocol recommended ADT administration in accordance with NCCN and EAU guideline recommendations [16], [17]. Accordingly, short-term ADT should be added for uIR patients, but not for favorable intermediate-risk and low risk patients. ADT was administered in 35.4% of patients with favorable intermediate-risk disease and 63.8% of patients with unfavorable intermediate-risk disease, indicating substantial variation in treatment patterns across risk groups.

The relatively frequent use of ADT in favorable intermediate risk disease may reflect referring physician preference and likely influenced both early PSA kinetics and sexual quality-of-life outcomes. As illustrated by Fig. 3C ADT was associated with substantially lower sexual quality-of-life scores throughout follow-up, whereas patients treated with UHFX without ADT experienced only a modest decline. These findings highlight the importance of appropriate patient selection for ADT and suggest that avoiding unnecessary ADT exposure may reduce treatment-related decline of sexual quality of life in patients with low- and favorable intermediate-risk disease. Overall, these findings illustrate the variability of UHFX implementation during the early adoption phase of prostate SBRT.

In comparison with PACE-B, grade 1–2 GU toxicity at treatment completion was 73.7% in the present cohort and 94.7% in PACE-B, while grade ≥ 3 GU toxicity remained below 1% in both studies. Grade 1–2 GI toxicity was 16.7% in the present registry compared with 73% in PACE-B, with low rates of grade ≥ 3 GI toxicity in both cohorts [9].

Comparable findings were reported in the recently published PACE-C trial, which included patients with unfavorable intermediate- and high-risk disease receiving concomitant ADT [13]. At 12 weeks, PACE-C reported grade ≤ 2 GU toxicity of 66% and GI toxicity of 26%, while severe toxicity remained uncommon. A detailed comparison of toxicity outcomes between studies is provided in Table 3.

**Table 3:** CTC graded toxicity comparison with PACE-B and C trials.

	Austrian registry		PACE-B		PACE-C	
	GI	GU	GI	GU	GI	GU
**G1** **+** **2**	18.4%	75.2%	73%	94.7%	26%	66%
**G3**	0%	0.6%	0.7%	1.7%	0%	0%

Patient-reported quality-of-life outcomes further support the tolerability of linac-based UHFX in routine clinical practice. Across urinary outcome measures, the greatest symptom burden was consistently detected immediately after completion of radiotherapy, followed by recovery during subsequent follow-up. This pattern was evident both in EPIC urinary domain scores and IPSS/IPSS-related quality-of-life assessments and mirrors the peak in acute physician-reported GU toxicity. In contrast, bowel-related quality of life remained largely stable throughout follow-up, corresponding to the low rates of GI toxicity observed clinically. Sexual function appeared to be influenced predominantly by ADT exposure rather than to radiotherapy alone, highlighting the importance of considering systemic treatment effects when interpreting quality-of-life outcomes.

The limitations of the present study include its non-randomized single-arm design and the absence of fully standardized treatment protocols across participating centers. In addition, lower-grade toxicities may have been underreported, which represents a recognized limitation of registry-based analyses compared with randomized clinical trials. The median follow-up remains insufficient to assess late toxicity and long-term biochemical control. Furthermore, short term ADT influences PSA kinetics and thus masks the true PSA response to UHFX in the subgroup of patients who have received ADT. Continued follow up will be necessary to evaluate long term toxicity and oncologic endpoints such as biochemical free survival and metastases free survival. Despite these limitations, the registry offers valuable insight into current UHFX practice across heterogeneous national treatment sites and reflects the early integration of linac-based SBRT into routine clinical care.

## 5 Conclusion

These findings support UHFX as a safe and effective treatment approach for localized prostate cancer in routine clinical practice. Early oncologic results and toxicity profiles were consistent with those reported in previous randomized trials. Continued follow-up is needed to assess long-term toxicity and oncologic outcomes.

The following are the supplementary data related to this article.

Supplementary material

## CRediT authorship contribution statement

**Natalee Annon-Eberharter:** Writing – review & editing, Project administration, Investigation, Formal analysis, Data curation. **Johannes Berchtold:** Methodology, Formal analysis. **Angela Ginestet:** Investigation. **Georg Gruber:** Investigation. **Lukas Kocik:** Investigation. **Oxana Komina:** Investigation. **Tanja Langsenlehner:** Investigation. **Elisabeth Nechvile:** Investigation. **Christoph Resl:** Investigation. **Falk Röder:** Writing – review & editing, Resources, Investigation. **Samuel M. Vorbach:** Investigation. **Ludmila Zahlbruckner:** Investigation. **Frank Wolf:** Writing – review & editing, Writing – original draft, Supervision, Methodology, Conceptualization.

## Declaration of competing interest

The authors declare the following financial interests/personal relationships which may be considered as potential competing interests:

Falk Roeder reports a relationship with GSK, BMS (grant for ABCSG projects) that includes: funding grants. Falk Roeder reports a relationship with CRAD, Siemens Healthineers, IntraOP (reference center contracts) that includes: funding grants. Falk Roeder reports a relationship with Dr. Falk Pharma (study participation - case fees) that includes: funding grants. Falk Roeder reports a relationship with Johnson and Johnson, MSD, Pharmamar, IntraOP, Raysearch that includes: consulting or advisory, speaking and lecture fees, and travel reimbursement. ESTRO Guideline committee subgroup GI: lead Landesfachgruppe Radioonkologie Salzburg: lead Head of Department of Radiation Oncology PMU Salzburg Head of Institute of Research and Development on Advanced Radiation Technologie (radART) Board member ABCSG colorectal branch If there are other authors, they declare that they have no known competing financial interests or personal relationships that could have appeared to influence the work reported in this paper.
