# Supplementary material for: Ultrahypofractionated linac-based radiotherapy of primary prostate cancer - early results of an Austrian nationwide registry study
Source: Clin Transl Radiat Oncol. 2026 Jul 21;61:101242. doi: 10.1016/j.ctro.2026.101242 (PMC13487599; doi:10.1016/j.ctro.2026.101242)
Supplement: Supplementary file 1 [file mmc2.docx]

| **Timepoint** | **Eligible patients** | **EPIC-26 completed** | **EPIC-26 return rate** | **IPSS completed** | **IPSS return rate** |
| --- | --- | --- | --- | --- | --- |
| baseline | 169 | 155 | 91.7% | 155 | 91.7% |
| RT end | 160 | 145 | 90.6% | 143 | 89.4% |
| 3 Monate | 135 | 128 | 94.8% | 126 | 93.3% |
| 6 Monate | 125 | 123 | 98.4% | 117 | 93.6% |
| 12 Monate | 78 | 78 | 100% | 75 | 96.2% |

Supplementary Table S1. Compliance With Longitudinal Patient-Reported Outcome Assessment

|  | **Timepoint** | **n** | **Median** | **Q1** | **Q3** |
| --- | --- | --- | --- | --- | --- |
| EPIC-26 Urinary | Baseline | 154 | 87.5 | 76.58 | 97.92 |
|  | End of RT | 142 | 68.75 | 55.08 | 87.5 |
|  | 3 months | 124 | 87.5 | 76.42 | 97.92 |
|  | 6 months | 122 | 89.58 | 78.5 | 100 |
|  | 12 months | 78 | 93.08 | 79.35 | 100 |
| EPIC-26 Bowel | Baseline | 152 | 100 | 91.67 | 100 |
|  | End of RT | 139 | 95.83 | 75 | 100 |
|  | 3 months | 124 | 100 | 91.67 | 100 |
|  | 6 months | 121 | 100 | 91.67 | 100 |
|  | 12 months | 78 | 100 | 91.67 | 100 |
| EPIC-26 Sexual | Baseline | 123 | 48.67 | 22.17 | 79.17 |
|  | End of RT | 112 | 29.84 | 20.83 | 66.62 |
|  | 3 months | 104 | 40.33 | 16.67 | 61.5 |
|  | 6 months | 102 | 34.67 | 16.67 | 70.5 |
|  | 12 months | 69 | 40.33 | 20.83 | 61.17 |
| IPSS Score | Baseline | 154 | 6 | 3 | 10 |
|  | End of RT | 141 | 13 | 7 | 18 |
|  | 3 months | 123 | 7 | 5 | 10.5 |
|  | 6 months | 117 | 7 | 4 | 11 |
|  | 12 months | 75 | 5 | 3 | 10 |
| IPSS QOL Index | Baseline | 145 | 1 | 1 | 2 |
|  | End of RT | 138 | 2 | 1 | 3 |
|  | 3 months | 123 | 1 | 1 | 2 |
|  | 6 months | 121 | 1 | 1 | 2 |
|  | 12 months | 74 | 1 | 1 | 2 |

Supplementary Table S2. EPIC-26 domain scores and IPSS scores for each time-point with interquartile ranges Q1 and Q3
